# Knowledge of obstetric fistula among prenatal clinic attendees and midwives in Mfantsiman municipality, Ghana

**DOI:** 10.1002/ijgo.13034

**Published:** 2020-01-13

**Authors:** Wisdom K. Azanu, Edward T. Dassah, Evans K. Agbeno, Anthony A. Ofori, Henry S. Opare‐Addo

**Affiliations:** ^1^ School of Medicine University of Health and Allied Sciences Ho Ghana; ^2^ School of Public Health Kwame Nkrumah University of Science and Technology Kumasi Ghana; ^3^ Department of Obstetrics and Gynecology Komfo Anokye Teaching Hospital Kumasi Ghana; ^4^ School of Medical Sciences University of Cape Coast Cape Coast Ghana; ^5^ School of Medicine and Dentistry Kwame Nkrumah University of Science and Technology Kumasi Ghana

**Keywords:** Midwives, Obstetric fistula, Obstructed labor, Prenatal attendees, Prolonged labor, Supervised delivery

## Abstract

**Objective:**

To determine obstetric fistula knowledge among prenatal attendees and midwives in Mfantsiman municipality, Ghana.

**Methods:**

An analytical cross‐sectional study was conducted among prenatal clinic attendees and midwives in Mfantsiman municipality from March to April, 2016. Women were selected by systematic sampling and consenting midwives were recruited. Respondents were interviewed using a pretested structured questionnaire. Data were analyzed using the χ^2^ test and Poisson regression with a robust error variance to generate relative risks (RRs) with 95% confidence intervals (CIs). *P*<0.05 was considered statistically significant.

**Results:**

Altogether, 393 prenatal attendees and 45 midwives were studied. Mean age of attendees was 28.1 ± 7.1 years. About 29% of prenatal attendees knew of, 37.2% had poor knowledge of, and 56.6% had some misconceptions about obstetric fistula. Women who had attained some level of education (*P* trend=0.001), were employed (adjusted RR 4.92; 95% CI, 1.98–12.21), or had given birth before (*P* trend=0.01) were more likely to have heard of obstetric fistula. All midwives knew of obstetric fistula and its preventive measures; however, up to 73.3% had some misconceptions about it.

**Conclusion:**

Educating prenatal attendees and organizing regular refresher courses on obstetric fistula for midwives should be a priority in the municipality.

## Abstract 2

Educating prenatal attendees and organizing regular refresher courses on obstetric fistula for midwives should be a priority in Mfantsiman municipality.

## 1 INTRODUCTION

Obstetric fistula is a complication that arises from prolonged and obstructed labor without prompt medical care.^1^ It puts considerable strain on the health of affected women as a result of associated fecal and/or urinary incontinence. Obstetric fistula is largely confined to low‐resource countries owing to inadequate utilization or nonavailability of maternity services.^2^, ^3^ At a fistula hospital in Ethiopia, over 90% of fistula cases occurred after prolonged (duration of labor more than 1 day) or obstructed labor; the stillbirth rate was also over 90%.^4^ The World Health Organization (WHO), estimates that between 50 000 and 100 000 women develop obstetric fistula each year and over 2 million women currently live with the condition. An incidence rate as high as 10 per 1000 births has been reported in sub‐Saharan Africa.^1^ A recent survey estimated the annual incidence of obstetric fistula in Ghana to be 1.6–1.8 per 1000 deliveries, with the Central Region having one of the highest incidence rates.^5^ These are largely deemed to be underestimates, as most cases go unreported owing to the associated stigma.^1^

Access to a skilled birth attendant at delivery and emergency obstetric care are critical for preventing obstetric fistula. Although prenatal clinic attendance is very high in Ghana (97% for at least one visit), the number of deliveries conducted by skilled birth attendants is much lower (74%).^6^ Up to 30% of deliveries in the Central Region (where the study was conducted) take place outside a health facility despite high prenatal care attendance rates.^6^ Supervised delivery rates could be improved if expectant mothers understood the complications associated with prolonged and obstructed labor, including fistula. There are limited data on knowledge of obstetric fistula among prenatal clinic attendees and healthcare providers in communities in Ghana where the condition is prevalent, such as Mfantsiman municipality. This municipality has the highest prevalence of obstetric fistula cases in the Central Region. The aim of the present study was to assess knowledge of obstetric fistula among prenatal clinic attendees and midwives in Mfantsiman municipality.

## 2 MATERIALS AND METHODS

An analytical cross‐sectional study was conducted among prenatal clinic attendees and midwives at the two main hospitals (District and Mercy Women's Hospital) and all five health centers in Mfantsiman municipality, Ghana, from March 15 to April 30, 2016.

Mfantsiman municipality is located on the Atlantic coast of the Central Region, comprising around 300 km^2^. Its administrative capital is Saltpond. The municipality has a total population of 144 332, of which 55% is female; approximately 50% (39 402) of the female population is of reproductive age (15–49 years).^7^ Nearly two‐thirds (65%) of the population live in urban localities and the remaining 35% live in rural areas.^7^

The primary level of care in Ghana has three sublevels: community, health center, and district hospital. The district hospital is the first referral point and handles many more patients than the two other sublevels. The municipality has five subdistricts, each of which has a health center. The two main hospitals in the municipality are the District Hospital at Saltpond and Mercy Women's Hospital at Mankessim. The latter has a fistula center. There were 33 regular midwives with 12 additional rotation midwives (national service personnel) in the municipality at the time of study, making a total of 45 midwives.

All prenatal clinic attendees and midwives in the municipality within the study period were eligible for inclusion in the study. Expectant mothers or midwives who declined consent to participate were excluded. Prenatal attendees with obstetric complications were also excluded.

The study was approved by the Committee on Human Research, Publication, and Ethics (CHRPE) of Kwame Nkrumah University of Science and Technology. Participation in the study was entirely voluntary and informed consent was obtained from each woman. For minors (girls under 18 years of age), informed consent and assent were obtained from the parent/guardian and the woman, respectively.

### 2.1 Sample size estimation

An estimated sample size of 390 had adequate power of 80% to detect knowledge of obstetric fistula among prenatal clinic attendees in the municipality, assuming the proportion of attendees with knowledge of obstetric fistula in the municipality was similar to the 36% reported in Burkina Faso.^3^ The number of women recruited from each facility was calculated in proportion to the reported prenatal care attendance for the first half of 2015. All 45 midwives in the municipality were invited to participate in the study.

### 2.2 Sampling and procedure for data collection

Eligible women were selected by systematic sampling using the prenatal clinic attendance list for each day as the sampling frame. The sampling interval (*x*) for each day was obtained by dividing the number of attendees to be recruited that day by the total number (*N*) of eligible attendees at the clinic that day. The first case (*y*) was selected by simple random sampling through balloting. The rest of the participants were then obtained by *y *+ *x*,* y* + 2*x*,* y* + 3*x*, etc. Where a selected woman was not available at the time of the interview or declined consent, the next eligible woman on the attendance list for that day was selected.

Eligible women were approached individually by a member of the research team who explained the purposes and benefits of the study and obtained informed consent. Consenting attendees and midwives were interviewed individually and data collected on their sociodemographic and reproductive characteristics, and knowledge of obstetric fistula using a pretested structured questionnaire.

Knowledge of obstetric fistula was assessed by evaluating responses to 11 questions on fistula including the causes, risk factors, and prevention of obstetric fistula. Each correct answer was assigned a score of +1 while each incorrect or undecided (“don't know”) response attracted a score of 0. The scores for each woman were summed and categorized.

### 2.3 Data analysis

Data were double entered into Epi Info version 7.1.1.14 (CDC, Atlanta, GA, USA) and exported to Stata version 12.0 (Stata Corp, College Station, TX, USA) for analysis. The knowledge scores for each woman were categorized as follows: 0–3 (low); 4–6 (average); and 7–11 (high). Women with low scores were considered to have poor knowledge while those with average or high scores were considered to have good knowledge of obstetric fistula. Data were summarized using descriptive statistics and charts. Categorical variables were compared using the χ^2^ or Fisher exact test as appropriate. Factors associated with awareness of obstetric fistula among prenatal clinic attendees were assessed by calculating crude and adjusted relative risks (RRs) with corresponding 95% confidence intervals (CIs) using univariate and multivariate Poisson regression with a robust error variance. *P*<0.05 was considered statistically significant.

## 3 RESULTS

A total of 393 prenatal clinic attendees and all 45 midwives in the municipality were recruited into the study. The midwives were of different categories, comprising 12 rotation midwives, 26 staff midwives, two senior staff midwives, and five midwifery officers. The mean age of the clinic attendees was 28.1 ± 7.1 years (range, 15–48 years). Nearly half (n=189, 48%) were in their 20s and about 11% (n=44) were adolescents. Over 90% (n=360) of the women had had some formal education, with the majority completing basic education. About 80% (n=304) were married/cohabiting or employed (n=314). Median parity was 1 (interquartile range, 1–3). Half of the women were multiparous. About 60% (n=240) of the expectant mothers received prenatal care in hospitals and nearly 40% (n=153) in health centers (Table 1).

**Table 1:** Sociodemographic characteristics and type of health facility attended by women attending prenatal care clinics in Mfantsiman municipality

Variable	No. (%) (n=393)
Age, y	
15–19	44 (11.2)
20–29	189 (48.1)
≥30	160 (40.7)
Educational level^a^	
No formal education	32 (8.2)
Basic education	252 (64.3)
Secondary and higher education	108 (27.5)
Marital status^a^	
Single	88 (22.4)
Married/cohabiting	304 (77.6)
Occupation	
Unemployed	79 (20.1)
Semiskilled	272 (69.2)
Skilled	42 (10.7)
Parity	
0	77 (19.6)
1	120 (30.5)
≥2	196 (49.9)
Type of facility	
Health center	153 (38.9)
Hospital	240 (61.1)

a Missing data from one (0.3%) attendee.

Less than one‐third (n=113, 28.8%) of the women had heard of obstetric fistula, with most obtaining their information from a health facility (41.6%) or family/friends (31.9%). Factors associated with their awareness of obstetric fistula are shown in Table 2. On univariate analysis, all sociodemographic characteristics were significantly associated with awareness of obstetric fistula. On multivariate analysis, educational level, occupation, and parity remained significantly associated with awareness of the condition. There was an increasing trend toward awareness of obstetric fistula and educational level (adjusted RR 2.45; 95% CI, 1.12–5.34; *P* trend=0.001). Being in skilled employment increased the odds of having heard of obstetric fistula nearly five‐fold (adjusted RR 4.92; 95% CI, 1.98–12.21) relative to being unemployed/housewife. Awareness of fistula increased with parity (adjusted RR 1.86; 95% CI, 1.13–3.09; *P* trend=0.01).

**Table 2:** Factors associated with awareness of obstetric fistula among women attending prenatal care clinics in Mfantsiman municipality

Variable	Awareness of obstetric fistula (n=113)		
	No. (%)	Crude RR (95% CI)	Adjusted RR (95% CI)
Age, y		*P*=0.06; *P* trend=0.01	*P*=0.71; *P* trend=0.46
15–19	6 (13.6)	1	1
20–29	53 (28.0)	2.06 (0.94–4.48)	0.77 (0.36–1.68)
≥30	54 (33.8)	2.48 (1.14–5.38)	0.72 (0.32–1.61)
Educational level		*P*<0.001; *P* trend<0.001	*P*<0.001; *P* trend=0.001
No formal education	6 (18.8)	1	1
Basic education	50 (19.8)	1.06 (0.49–2.27)	1.18 (0.55–2.52)
Secondary and higher education	57 (52.8)	2.81 (1.34–5.92)	2.45 (1.12–5.34)
Marital status		*P*=0.002	*P*=0.26
Single	12 (13.6)	1	1
Married/cohabiting	101 (33.2)	2.44 (1.45–4.22)	1.42 (0.77–2.59)
Occupation		*P*<0.001	*P*<0.001
Unemployed	6 (7.6)	1	1
Semiskilled	75 (27.6)	3.63 (1.64–8.03)	2.79 (1.18–6.60)
Skilled	32 (76.2)	10.0 (4.56–22.07)	4.92 (1.98–12.21)
Parity		*P*=0.02; *P* trend=0.01	*P*=0.02; *P* trend=0.01
0	14 (18.2)	1	1
1	31 (25.8)	1.42 (0.81–2.50)	1.25 (0.76–2.07)
≥2	68 (34.7)	1.91 (1.14–3.18)	1.86 (1.13–3.09)
Type of facility		*P*=0.37	—
Health center	40 (26.1)	1	—
Hospital	73 (30.4)	1.16 (0.84–1.62)	—

Of the 113 expectant mothers who had heard of obstetric fistula, 42 (37.2%) had poor knowledge, 71 (62.8%) had good knowledge, and 64 (56.6%) had misconceptions about the causes/risk factors for the condition. The perceived risk factors for obstetric fistula included home delivery (n=91, 80.5%), prolonged labor (n=76, 67.3%), teenage pregnancy and delivery (n=57, 50.4%), evil spirits (n=35, 31%), and sexually transmitted infections and improper use of reproductive health services such as family planning (n=84, 74.3%). The relationships between poor knowledge or misconceptions and sociodemographic characteristics and type of facility where women received prenatal care are shown in Table 3. Poor knowledge of the risk factors was associated with lower educational background (*P*=0.03), being single (*P*=0.004), or being unemployed (*P*=0.01). Women who received prenatal care in hospitals were more likely to have both poor knowledge (25% vs 43.8%; *P*=0.05) and misconceptions (24.4% vs 77.9%; *P*<0.001) about the risk factors for obstetric fistula compared with women who received care in health centers.

**Table 3:** Sociodemographic characteristics and poor knowledge and misconceptions among women attending prenatal care clinics who had heard of obstetric fistula (n=106)

Variable	Poor knowledge (n=42)		Misconceptions (n=64)	
	No. (%)	*P* value	No. (%)	*P* value
Age, y		0.64		0.51
15–19	3 (50.0)		5 (71.4)	
20–29	21 (39.6)		34 (64.2)	
≥30	18 (33.3)		25 (47.2)	
Educational level		0.03		0.69
No formal education	4 (66.7)		3 (42.9)	
Basic education	23 (46.0)		30 (55.6)	
Secondary and higher education	15 (26.3)		31 (59.6)	
Marital status		0.004		0.46
Single	9 (75.0)		8 (66.7)	
Married/cohabiting	33 (32.7)		56 (55.5)	
Occupation		0.01		0.38
Unemployed	4 (66.7)		4 (57.1)	
Semiskilled	33 (40.0)		41 (52.6)	
Skilled	5 (15.6)		19 (67.9)	
Parity		0.14		0.36
0	4 (28.6)		9 (75.0)	
1	16 (51.6)		19 (57.6)	
≥2	22 (32.4)		36 (52.9)	
Type of facility		0.05		<0.001
Health center	10 (25.0)		11 (24.4)	
Hospital	32 (43.8)		53 (77.9)	

On the availability of treatment for obstetric fistula, two‐thirds (n=74, 66.7%) of the women who knew about obstetric fistula agreed that the condition could be treated within the country. About three‐quarters (n=76, 76%) considered surgery to be the treatment modality of choice. Most women (n=103, 92.8%) agreed that supervised delivery by trained health workers could help prevent obstetric fistula.

All midwives were aware of obstetric fistula. The majority (n=39, 86.7%) of midwives had good knowledge, whereas 6 (13.3%) had poor knowledge. Almost three‐quarters of midwives (n=33, 73.3%) had some misconception about obstetric fistula. Almost all (n=43, 95.6%) considered home delivery to be a risk factor for the condition and also agreed that evil spirits were not a cause (n=42, 93.3%). Most (n=41, 91.1%) did not associate obstetric fistula with bad luck. However, as many as 73.3% (n=33), 53.3% (n=24), and 40% (n=18) of midwives considered induced abortion, sexually transmitted infections, and improper use of family planning, respectively, to be risk factors for obstetric fistula. All midwives knew that surgery was the treatment modality of choice and that patients had to be referred to the fistula hospital for the surgery. Regarding prevention of the condition, 100% of midwives agreed that delivery by a trained health professional or undergoing cesarean delivery when indicated could help prevent occurrence of fistula. The 43 (95.6%) midwives who answered the question on the partograph believed that use of the partograph could prevent obstructed labor.

## 4 DISCUSSION

Less than one‐third of prenatal care clinic attendees had heard of obstetric fistula, nearly 40% of whom had poor knowledge and over half had misconceptions about its risk factors. Significant factors associated with being aware of the condition were educational background, occupation, and parity. Poor knowledge was associated with lower educational level, being single, unemployed, or receiving prenatal care in a hospital. Although most midwives were knowledgeable of the risk factors for obstetric fistula, up to three‐quarters associated some basic reproductive health services with its occurrence.

The less than 30% rate of awareness in this study is comparable to the low rates reported in most countries in sub‐Saharan Africa.^3^, ^8^ However, it is lower than the 45% awareness rate reported in Northern Ghana,^9^ and 44%–61% in other parts of Africa.^10^, ^11^, ^12^ The higher awareness rate observed in some of the previous studies may be attributed to prior educational campaigns on obstetric fistula and conducting the study among fistula patients or participants with prior exposure to information on the condition.^9^, ^11^, ^12^ For example, in Northern Ghana where fistula is relatively common, more educational campaigns are directed toward these populations. These may have accounted for the higher level of awareness of obstetric fistula in Northern Ghana.^9^

Less than half of the women who had heard of fistula in our study obtained their information from the health facility or healthcare provider, suggesting that discussions on fistula may not have been an integral component of prenatal care in most facilities in the municipality. It is also conceivable that information on fistula provided by healthcare providers may not have been very accurate as the majority of these providers had some misconceptions about the condition. Owing to the much smaller number of patients seeking care at health centers, they tend to have better health provider–patient contacts and interactions. This probably explains why expectant mothers who received care in the health centers had better knowledge of obstetric fistula compared with their counterparts in the hospitals.

Knowledge of the causes and risks factors for obstetric fistula among women who had heard of the condition could generally be described as low. These findings are consistent with those of a recent nationwide survey in which obstetric fistula was commonly attributed to adultery, sorcery, evil spirits, or bad luck.^5^ Similar findings have been reported across Africa.^2^, ^10^, ^12^ These misconceptions have implications for the uptake of reproductive health services, which is worrisome. For instance, should some of these women develop obstetric fistula, they may not seek treatment from the hospitals, but rather go to so‐called spiritual healing centers. More than half of the women associated obstetric fistula with reproductive tract conditions and services, which could undoubtedly affect the use of family planning services by these women. It is, however, quite reassuring that about half of the respondents could identify teenage pregnancy and delivery as a risk factor for developing fistula, as was observed in Nigeria.^13^

Women in employment are more likely to seek prenatal care services as they are less likely to face financial barriers. In addition, parous women may have had more prenatal care visits cumulatively, thus increasing their chances of hearing about obstetric fistula. Consistent with the results of previous studies in Burkina Faso^3^ and Kenya,^12^ we observed a trend toward increasing knowledge of obstetric fistula and higher educational level. There is some evidence that education, even at the basic level, reduces gaps in knowledge about fistula and prevents its occurrence, empowers women, and ultimately improves their health outcomes.^3^, ^14^ This emphasizes the need to improve intersectoral collaboration between the ministries of education and health to optimize health outcomes for women, especially in low‐resource countries like Ghana.

Surprisingly, the majority of midwives also had misconceptions about fistula; for example, most midwives attributed obstetric fistula to sexually transmitted infections or improper use of family planning methods. These misconceptions are worrying because one would expect that midwives, having received some level of training, should not be influenced by existing myths in the communities about obstetric fistula. These observations may point to deficiencies in the preservice training curricula (especially if these midwives were trained within the municipality) or expected competencies during clinical/practical rotations. Moreover, this is a district where fistula cases are managed regularly and information on it should not only be readily available, but healthcare providers should be abreast of such information as they are likely to be the first points of contact within their communities and facilities. Fortunately, almost all midwives recognized the partograph as a tool for preventing obstructed labor. Furthermore, all midwives knew that surgical correction was the treatment modality of choice and knew where to refer such patients for surgery. This is commendable and good for medical care as patients who present with symptoms of obstetric fistula are likely to be referred for the appropriate treatment.

The study has some limitations. It was conducted in an area with a fistula center, therefore awareness of obstetric fistula in this population is likely to be relatively higher and should not be extrapolated to the general population. In addition, women who did not attend prenatal care were excluded. Therefore, the findings of this hospital‐based study cannot be generalized to the entire population of pregnant women in the municipality. Finally, the number of midwives in the municipality was relatively small making it difficult to compare levels of knowledge of obstetric fistula across the different categories.

In conclusion, awareness of obstetric fistula among prenatal care clinic attendees in this municipality with a fistula center is relatively low and most attendees had some misconceptions regarding the condition's risk factors. Although midwives generally had good knowledge, some had misconceptions, indicating a gap between midwives’ knowledge and perception. Therefore, there is an urgent need to educate women more regularly on obstetric fistula during prenatal care. Regular refresher courses on obstetric fistula should also be organized for midwives to update their knowledge and help dispel some of their misconceptions.

## AUTHORS CONTRIBUTIONS

WKA, ETD, EKA, AAO, and HSOA conceived and designed the study. WKA collected the data. WKA and ETD analyzed the data. All authors read and approved the final manuscript.

## CONFLICTS OF INTEREST

The authors have no conflicts of interest.
